# Recent Advances in Electrospun Nanofiber-Based Self-Powered Triboelectric Sensors for Contact and Non-Contact Sensing

**DOI:** 10.3390/nano15141080

**Published:** 2025-07-11

**Authors:** Jinyue Tian, Jiaxun Zhang, Yujie Zhang, Jing Liu, Yun Hu, Chang Liu, Pengcheng Zhu, Lijun Lu, Yanchao Mao

**Affiliations:** Key Laboratory of Materials Physics of Ministry of Education, School of Physics and Microelectronics, Zhengzhou University, Zhengzhou 450001, China

**Keywords:** electrospun nanofibers, triboelectric nanogenerators, self-powered sensors, contact and non-contact sensing

## Abstract

Electrospun nanofiber-based triboelectric nanogenerators (TENGs) have emerged as a highly promising class of self-powered sensors for a broad range of applications, particularly in intelligent sensing technologies. By combining the advantages of electrospinning and triboelectric nanogenerators, these sensors offer superior characteristics such as high sensitivity, mechanical flexibility, lightweight structure, and biocompatibility, enabling their integration into wearable electronics and biomedical interfaces. This review presents a comprehensive overview of recent progress in electrospun nanofiber-based TENGs, covering their working principles, operating modes, and material composition. Both pure polymer and composite nanofibers are discussed, along with various electrospinning techniques that enable control over morphology and performance at the nanoscale. We explore their practical implementations in both contact-type and non-contact-type sensing, such as human–machine interaction, physiological signal monitoring, gesture recognition, and voice detection. These applications demonstrate the potential of TENGs to enable intelligent, low-power, and real-time sensing systems. Furthermore, this paper points out critical challenges and future directions, including durability under long-term operation, scalable and cost-effective fabrication, and seamless integration with wireless communication and artificial intelligence technologies. With ongoing advancements in nanomaterials, fabrication techniques, and system-level integration, electrospun nanofiber-based TENGs are expected to play a pivotal role in shaping the next generation of self-powered, intelligent sensing platforms across diverse fields such as healthcare, environmental monitoring, robotics, and smart wearable systems.

## 1. Introduction

In recent years, the demand for sustainable, miniaturized and autonomous energy solutions has been constantly increasing, which has accelerated the global efforts in the development of innovative energy harvesting technologies [1]. The Internet of Things, wearable electronic products, and biomedical devices have all developed rapidly, which has exposed some restrictions of traditional battery-powered platforms, such as limited energy density, relatively bulky forms, environmental pollution, and the need for frequent charging or battery replacement [2]. These limitations have hindered the long-term deployment and scalability of next-generation electronic systems [3]. To address these challenges, self-powered energy conversion systems that can sustainably obtain energy from the environment and supply power to low-energy-consuming devices have become a crucial research direction. Triboelectric nanogenerators (TENGs) have attracted much attention due to advantages such as their simple structure, relatively high energy conversion efficiency, the wide range of possible materials used in their production, and relatively low production cost. Since the concept was first proposed in 2012, TENGs have been widely explored in fields such as intelligent sensing, wearable electronics, environmental monitoring, robotics, and healthcare [4,5,6,7].

Although TENGS have developed quite rapidly, large-scale practical applications are still hindered by several technical challenges. These challenges include limited electrical output, mechanical instability, insufficient long-term durability, and the difficulty in integrating with flexible or stretchable substrates [8]. The key issues lie in the development of high-performance triboelectric materials and structural design. Only in this way can surface charges be generated and retained to the greatest extent [9,10]. The surface energy, dielectric constant, electron affinity and mechanical flexibility of the interface material, along with the appearance of the surface and the behavior of interface contact, all have a significant impact on the triboelectric output of a TENG. The triboelectric layer of the micro–nano structure can increase the effective contact area and triboelectric interaction, thereby significantly improving the efficiency of charge transfer [11]. Electrospinning technology has particular advantages in manufacturing nanostructured polymer fibers with customized surface features and ideal mechanical properties [12].

Electrospinning is a universal and scalable technology that can produce continuous nanofibers with diameters ranging from tens of nanometers to several micrometers [13]. These nanofibers have a high surface-to-volume ratio, a porous structure, a very light weight, and excellent mechanical flexibility, which are crucial characteristics for optimizing the performance of flexible TENGs. More importantly, electrospinning can incorporate a variety of materials, such as natural or synthetic polymers, nanoparticles, conductive fillers, and functional additives [14,15]. This facilitates the design of pure polymer and composite nanofibers. By carefully adjusting parameters such as solution concentration, voltage, flow rate, and collector design, the arrangement, orientation, and shape of nanofibers can be precisely controlled to meet specific tribological performance requirements. Integrating electrospun nanofibers can enhance the interface contact and output efficiency of TENGs, and also enable the creation of innovative device architectures, including stretchable, wearable and textile-based systems [16]. Electrospinning has become a key technology driving the development of the next generation of high-performance, self-powered triboelectric sensor devices [17,18].

**Figure 1:**
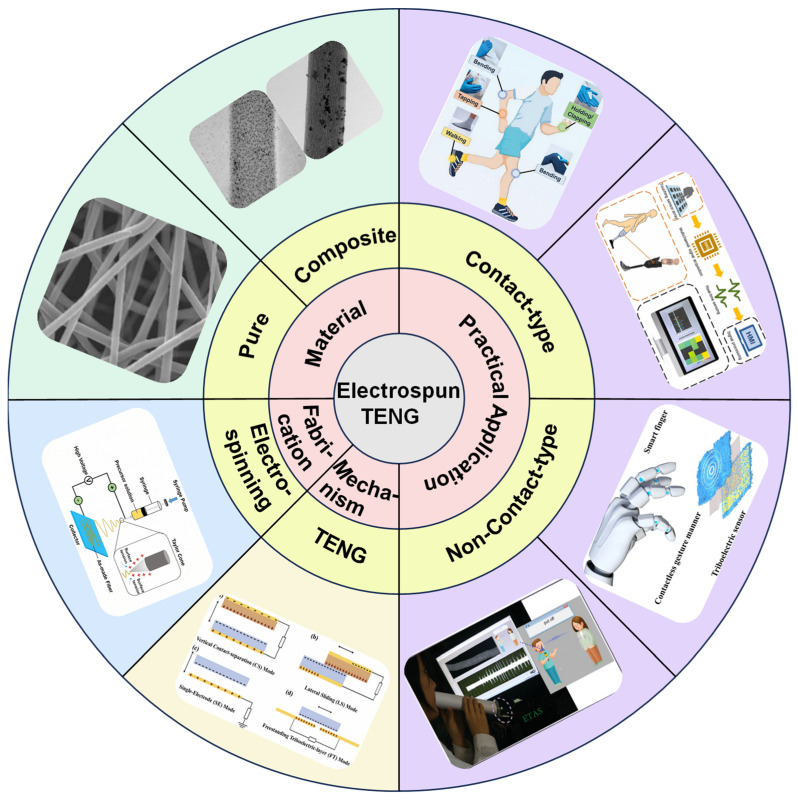
Overview of electrospun TENGs. Fabrication, working principle, materials, and practical applications.

This review aims to provide a comprehensive and systematic overview of recent advances in electrospun triboelectric nanogenerators (TENGs), with particular emphasis on fabrication methodologies, material design strategies, and their integration into practical self-powered sensing systems [19]. As shown in Figure 1, the discussion begins with a detailed explanation of the fundamental working principles of TENGs, followed by an introduction to the four primary operational modes of contact-separation, lateral-sliding, single-electrode, and freestanding triboelectric layer modes. Each is designed to harvest mechanical energy from various environmental sources [20]. Subsequently, we summarize the electrospinning technologies and key parameters that influence fiber morphology and device performance [21]. A comprehensive classification of electrospun materials is then presented, encompassing both pure polymer nanofibers and multifunctional composites enhanced with nanoparticles [22]. Recent progress in the application of electrospun TENGs for self-powered sensing is reviewed, focusing on both contact-type and non-contact-type scenarios, such as human–machine interaction, physiological monitoring, gesture recognition, and acoustic detection. Finally, we highlight existing challenges and provide insightful perspectives on future development directions in this rapidly evolving field [23,24,25].

## 2. Basic Principles and Working Mode of TENGs

The most important principles of TENG are contact charging and electrostatic induction [26]. When two materials with different electron affinity contact, they transfer charges according to their position in the triboelectric series, leaving opposite sides charged. As they separate due to pressing, dragging or sliding (physical motions), the separated charges develop a voltage potential difference, causing an imbalance of electric charge within a conductor. Consequently, the electrons begin to flow outwards through the surrounding circuitry in order to balance the created potential, which forms the alternating electrical current [27], converting various kinds of low-frequency mechanical stimulations as power sources into usable electrical energy [28]. Depending on the mechanical motion and electrode configuration, TENGs can operate in four fundamental modes: the vertical contact–separation (CS) mode, where two surfaces repeatedly contact and separate along the normal direction, generating periodic electrostatic potential variations [29]; the lateral sliding (LS) mode, involving in-plane sliding between surfaces at specific positions, leading to continuous charge redistribution [30]; the single-electrode (SE) mode, which requires only one active electrode connected to the load while the other triboelectric surface is grounded [31]; and the freestanding triboelectric-layer (FT) mode, where a mobile dielectric layer moves between two stationary electrodes, inducing potential differences due to asymmetric charge distribution [32,33,34]. Each mode has its own advantages and is suitable for different applications based on its characteristics.

### 2.1. Vertical Contact–Separation Mode

**Figure 2:**
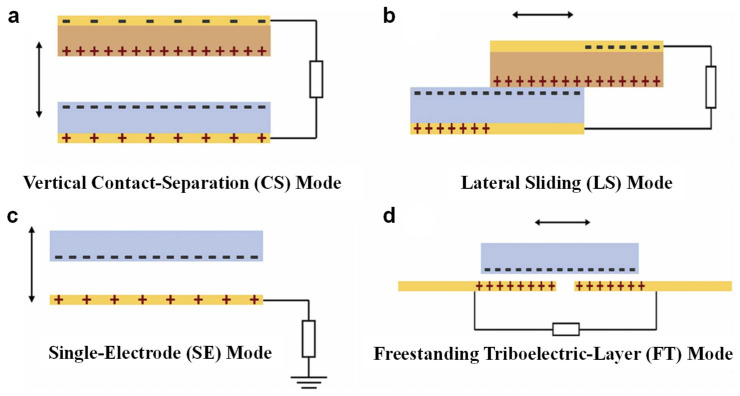
The four fundamental working mechanisms of triboelectric nanogenerators (TENGs). (**a**) Vertical mode based on contact and separation between two surfaces. (**b**) Mode involving lateral sliding motion between triboelectric layers. (**c**) Configuration utilizing a single active material with a grounded electrode. (**d**) Mode in which a freestanding triboelectric film moves between two stationary electrodes [31].

The vertical contact–separation (CS) mode is the most fundamental working mechanism of TENGs [35]. It involves two triboelectric materials with different electron affinities that periodically contact and separate in the direction perpendicular to the interface. Upon physical contact, opposite surface charges are generated due to triboelectrification [36]. When the materials are separated by an external mechanical force, an electric potential builds up between the electrodes, inducing electron flow in the external circuit to compensate for the electrostatic imbalance. As the cycle repeats, alternating current is produced (Figure 2a). Due to its simple structure and low interfacial friction, the CS mode is especially well-suited for applications that involve intermittent vertical contact, such as tactile sensing, footstep energy harvesting, and pulse monitoring. However, it is less suitable for environments that require continuous movement or compact system integration as maintaining an effective separation distance is crucial for its operation. Even with this constraint, the vertical contact–separation mode has been extensively adopted in both energy harvesting and pressure-sensitive technologies, owing to its ease of fabrication and consistent electrical performance.

### 2.2. Lateral Sliding Mode

In the lateral sliding (LS) mode, as shown in Figure 2b, two triboelectric layers undergo relative in-plane displacement induced by a shearing force, leading to continuous sliding between their surfaces [28]. This sliding alters the contact area dynamically, which disrupts the equilibrium of previously established charge centers, causing fluctuations in electrostatic potential. The resulting potential difference drives electrons through an external circuit, generating an alternating current. Compared to the vertical contact–separation mode, the LS mode supports more diverse mechanical configurations, such as rotary and linear-sliding structures, making it particularly suitable for harvesting energy from sustained directional motion. It is especially effective in systems like rotating machinery, rail-guided platforms, and human joints, where lateral displacement occurs repeatedly over time. However, the ongoing lateral friction may lead to increased mechanical wear, potentially affecting long-term device reliability. Despite this drawback, its directional insensitivity and mechanical adaptability make the LS mode a compelling choice for motion sensing and wearable electronics [37].

### 2.3. Single-Electrode Mode

As shown in Figure 2c, the single-electrode (SE) mode simplifies the TENG configuration by using only one active electrode, with the other side of the circuit connected to the ground. In this mode, the triboelectric material, which is often attached to a moving object, periodically approaches and departs from the stationary electrode [38]. Contact and separation between the material and the electrode result in charge transfer, inducing an electrostatic potential difference relative to the ground. This difference drives electrons between the electrode and ground through an external load. The SE mode is particularly advantageous in scenarios where direct wiring to both contact surfaces is impractical, such as in free-moving or body-attached systems. It is well-suited for applications requiring high mechanical flexibility and spatial freedom, including gesture recognition, human–machine interfaces, and self-powered touch panels, where only one accessible electrode is feasible [39]. Although its output performance is generally lower than that of dual-electrode configurations, the SE mode’s structural simplicity, lightweight design, and adaptability make it a popular choice for wearable electronics and interactive sensing platforms [40].

### 2.4. Freestanding Triboelectric-Layer Mode

The FT structure is shown in Figure 2d, where the dielectric layer runs back and forth between two unmovable symmetrical electrodes connected together by the circuit [41]. When moving laterally, the charge distribution changes with electrostatic charging/discharging on both sides. Changing the shift density induces a potential difference in the electrets, which drives current flow through the load. Unlike conventional contact-mode systems, the FT mode can generate electricity without direct mechanical contact at the electrode interface, making it less prone to mechanical failure and more suitable for long-term use. Its structural robustness allows for high-resolution sensing, especially when integrated with micropatterns or gratings to enhance spatial discrimination. The FT mode is particularly advantageous in applications demanding non-contact operation, high durability, and minimal maintenance, such as vibration detection, smart floor monitoring, and closed-environment sensing systems [42]. Furthermore, since there is no direct electrical connection between the moving object and the electrodes, this configuration significantly reduces mechanical wear, thereby offering enhanced longevity and improved reliability for detecting subtle micromotions [43]. Clearly, each of these modes provides many benefits depending upon its particular motion patterns and system structure. Proper understanding of these modes helps us make good use of them according to our different needs and application cases [44].

## 3. Electrospinning Techniques and Materials

### 3.1. Methods and Mechanisms of Electrospinning

Electrospinning is a versatile and efficient technique for producing polymer-based nanofibers (NFs) through the application of a high-voltage electrostatic field to a polymer solution or melt [45]. Unlike conventional fiber fabrication methods that rely on mechanical stretching or thermal gradients, electrospinning leverages electrostatic forces to elongate a viscoelastic jet into continuous fibers with diameters typically ranging from tens of nanometers to several micrometers [46]. This method enables the fabrication of nonwoven nanofibrous mats characterized by high surface-area-to-volume ratios, excellent porosity, and tunable morphological features that enhance the triboelectric performance of TENGs by increasing interfacial area and contact effectiveness [47,48].

**Figure 3:**
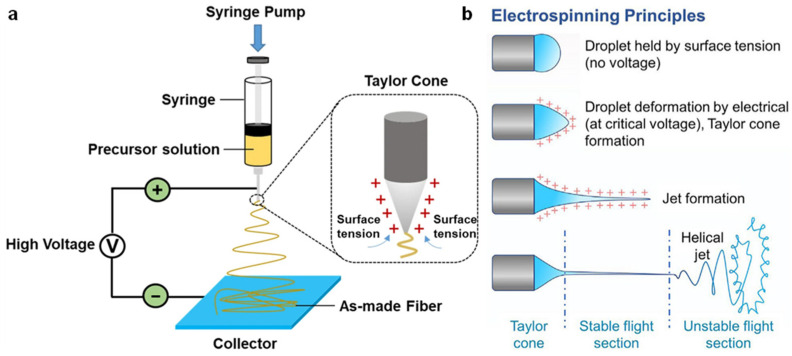
Overview of the electrospinning technique and its working mechanism for nanofiber fabrication. (**a**) Schematic illustration of a typical electrospinning setup [50]. (**b**) Mechanism of electrospinning showing droplet deformation, jet formation, and fiber deposition [56].

According to the study by Qiu et al., the electrospinning process involves applying a voltage, generally exceeding 5 kV, to a polymer solution dispensed from a spinneret (Figure 3a). When the electrostatic force acting on the charged fluid overcomes the liquid’s surface tension, a jet is ejected from the tip of the Taylor cone, initiating the fiber formation process [49]. As the charged jet travels toward a grounded collector, it undergoes rapid elongation, solvent evaporation, and solidification, ultimately forming nanofibers [50]. The resulting fiber morphology and diameter are influenced by multiple parameters, including solution viscosity, conductivity, surface tension, applied voltage, flow rate, and the distance between the spinneret and the collector, as well as ambient conditions such as temperature and humidity [51,52]. These parameters directly influence the nanofiber morphology, such as fiber diameter, porosity, and surface roughness, which in turn affect the TENG’s output behavior. Smaller fiber diameters typically lead to a higher surface-area-to-volume ratio, which enhances charge generation and collection efficiency by providing more surface area for contact. Additionally, increased porosity improves the flexibility and compressibility of the nanofibers, facilitating better contact with the counter electrode during operation, which directly impacts the efficiency of charge transfer. Furthermore, surface roughness influences the triboelectric interaction between the fibers and the counter electrode. A rougher surface can increase the mechanical contact area, leading to greater charge generation but may also introduce more frictional losses. Proper tuning of these parameters allows for the fabrication of nanofibers with optimized properties for high-performance energy conversion and sensing applications. These adjustments enable precise control over fiber architecture, including solid, porous, hollow, or core–shell structures, and the alignment (random or oriented) through modifications to the collector design [53]. Furthermore, the incorporation of high-molecular-weight polymers with low-molecular-weight ceramic precursors enhances viscosity, expanding the material versatility of electrospun TENGs [54,55].

Cho et al. conducted a detailed investigation into the dynamics of the electrospinning process, particularly focusing on the evolution of the polymer jet under an applied electrostatic field (Figure 3b). At the initial stage, the polymer solution forms a nearly spherical droplet at the spinneret tip, maintained by surface tension [57]. As the applied voltage increases [58], the electrostatic force gradually overcomes the surface tension, leading to the formation of a conical structure known as the Taylor cone [59]. Once the voltage surpasses a critical threshold, a charged liquid jet is emitted from the cone apex [56]. In contrast to simple electrospraying, which results in droplet dispersion, electrospinning is characterized by the viscoelastic behavior of the polymer solution and the degree of polymer chain entanglement. These rheological properties are essential for stabilizing the jet and ensuring the continuous formation of nanofibers [60]. As the charged jet extends, it initially follows a straight trajectory but soon undergoes a series of bending and whipping instabilities caused by coulombic repulsion between like-charged segments [61]. These instabilities elongate the jet, enhance solvent evaporation, and significantly reduce fiber diameter, ultimately resulting in the deposition of ultrafine [62], solidified nanofibers on the grounded collector surface [63].

However, when electrospinning technology is applied to the production of large-area triboelectric nanogenerators, significant challenges are encountered. In terms of the uniformity, mechanical stability, and reproducibility of nanofibers, a major issue is that it is particularly difficult to keep the diameter and shape of the fibers consistent over a wide range, features that are crucial for achieving improved triboelectric performance. The adoption of a multi-nozzle system can enhance uniformity, enabling the polymer solution to be distributed more evenly and the formation of fibers to be more consistent. Regarding the scalability issue, one solution is to use a roll-to-roll system, which can continuously produce nanofiber pads on large substrates, and these systems can keep electrospinning uniform across the entire width of the roller. During the continuous processing, there are still certain limitations in controlling the mechanical stability of fibers. This is because the electrospinning process is particularly sensitive to environmental factors such as temperature and humidity. Ensuring reproducibility between batches remains a significant challenge. Despite these challenges, recent progress has been made in process optimization and equipment design, which can help improve the scalability and reproducibility of large-scale electrospinning production.

Apart from electrospinning, other fiber manufacturing techniques, such as meltblown, template-assisted synthesis and centrifugal spinning, are also used to produce nanofibers for various purposes. Melt blowing is a process in which a polymer melt is extruded through a nozzle under high pressure, and the fibers thus obtained are rapidly cooled and then collected. This method has high scalability and relatively low production costs. However, it often has problems with a lack of fiber uniformity as it cannot well control the fiber morphology. In addition, template-assisted synthesis can produce fibers with precisely controllable diameters and structures, but because it requires a specific template and has to go through multiple processing steps, it may be more labor-intensive and have higher costs. Centrifugal spinning relies on a high-speed rotating disc to spin polymer solutions into fibers, which provides a fast and effective method for the production of nanofibers. However, it also faces challenges in terms of scalability and achieving uniform fiber performance over a wide range. Each method has its own specific advantages and challenges in terms of fiber uniformity, scalability, and cost-effectiveness [64]. Recent studies have also explored alternative materials for enhancing TENG performance, such as MXenes and polymer blends. MXenes are known for their high conductivity, mechanical strength, and flexibility, which make them a strong candidate for high-performance TENGs. Polymer blends, on the other hand, have been used to improve the mechanical properties and surface charge generation efficiency. However, electrospinning remains an effective method for fabricating nanofibers with precise control over morphology, which is crucial for optimizing triboelectric performance. Below is a table summarizing the key differences between electrospinning-based, MXenes-based, and polymer blend-based materials [65] (Table 1).

Overall, electrospinning remains a highly adaptable method that can be used to create one-dimensional nanostructures with customized properties, making it particularly suitable for high-performance triboelectric layers in nanogenerators [66]. By precisely controlling the morphology and arrangement of fibers, it can be optimized in various applications, such as energy harvesting and sensing. This also makes it different from other fiber manufacturing technologies [59].

### 3.2. Electrospun Materials

With the improvement in electrospinning techniques, researchers have reported many nanofiber materials suitable for different high-performance TENG application situations according to their needs. In this paper, we divide them into pure polymer nanofibers and composite polymer nanofibers [67], and summarize their diverse favorable properties such as morphology control, surface charge density, and functions that contribute to improving triboelectric output [68,69].

#### 3.2.1. Pure Polymer Nanofibers

**Figure 4:**
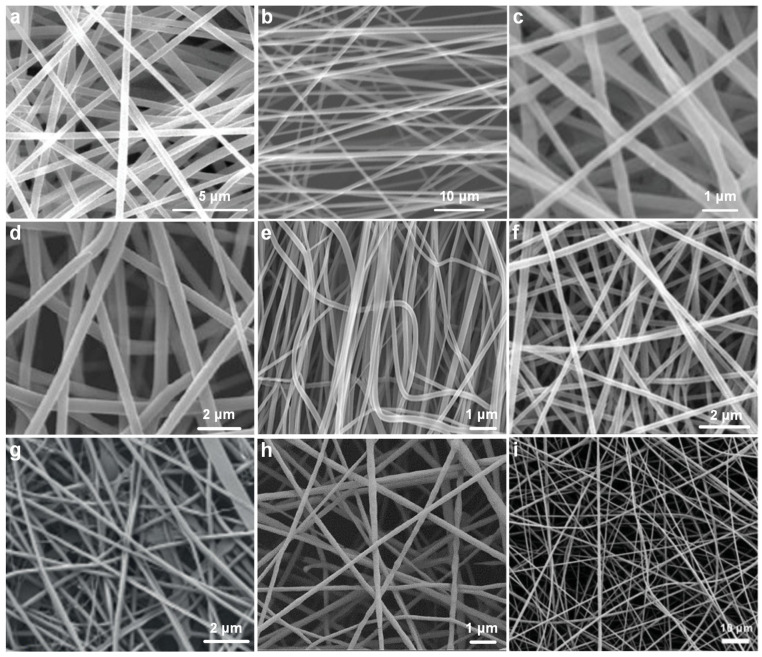
SEM images of electrospun pure polymer nanofibers. (**a**) PIL [72]. (**b**) PLA [73]. (**c**) PHBV [74]. (**d**) PAni [75]. (**e**) PAN [76]. (**f**) PA6 [77]. (**g**) Collagen [78]. (**h**) PCL [79]. (**i**) PVDF [80].

Pure polymer nanofibers not only exhibit excellent spinnability but also possess well-defined structures and tunable electrical properties, making them widely utilized in the fabrication of electrospun TENGs. Pure polymers can be precisely processed to form nanofiber membranes with a large specific surface area and uniform dielectric structure, which are critical for generating triboelectric charges through contact electrification and for effectively retaining the induced charges [70]. Nanofibers fabricated from several polymers commonly used in recent TENG research were characterized by SEM, as shown in Figure 4a–i. Each type of nanofiber exhibits distinct functional advantages depending on its material properties [71].

Poly(ionic liquid)s (PILs, Figure 4a), due to their inherent ionic conductivity and electrochemical stability, have demonstrated great potential for enhancing surface charge density and boosting triboelectric output [81]. Their ability to retain surface charges over extended periods makes them highly effective in applications requiring persistent electrical generation. Biodegradable polymers such as polylactic acid (PLA, Figure 4b) and poly(3-hydroxybutyrate-co-3-hydroxyvalerate) (PHBV, Figure 4c) offer a combination of environmental sustainability and mechanical flexibility [82]. These materials are especially attractive for transient electronics, wearable health monitoring devices, and green energy systems, where biocompatibility and disposability are required [83,84].

Conductive polymers such as polyaniline (Pani, Figure 4d) provide additional pathways for charge transport and can be strategically employed to enhance the interfacial charge transfer in hybrid systems [85]. Polyacrylonitrile (PAN, Figure 4e), recognized for its superior thermal stability and ease of electrospinning, serves as a reliable host matrix for further structural or compositional modifications. Nylon-6 (PA6, Figure 4f), with its robust mechanical strength and elasticity, supports applications involving repeated mechanical deformation, such as stretchable or wearable TENGs [86]. Naturally derived polymers such as collagen (Figure 4g) exhibit excellent biocompatibility and skin affinity, rendering them suitable for bio-integrated or epidermal electronics. Similarly, polycaprolactone (PCL, Figure 4h), another biodegradable polymer with favorable mechanical softness and a low melting point, is widely applied in biomedical and therapeutic devices [87]. Among these materials, polyvinylidene fluoride (PVDF, Figure 4i) stands out due to its exceptional electronegativity, ferroelectricity, and piezoelectricity [88]. These intrinsic properties enable PVDF-based nanofibers to generate significantly higher output performance in TENG devices, making it one of the most frequently employed polymers in high-efficiency energy harvesting applications [89].

Overall, the unique physicochemical properties of these pure polymers, such as the dielectric constant, mechanical flexibility, and surface morphology [90], can be effectively tailored through electrospinning process parameters including solution viscosity, applied voltage, needle-to-collector distance, and environmental humidity [91]. Furthermore, these polymers often serve as foundational matrices for subsequent compositional enhancements or structural engineering, which further expands their utility in multifunctional self-powered sensing systems [92].

#### 3.2.2. Composite Polymer Nanofibers

**Figure 5:**
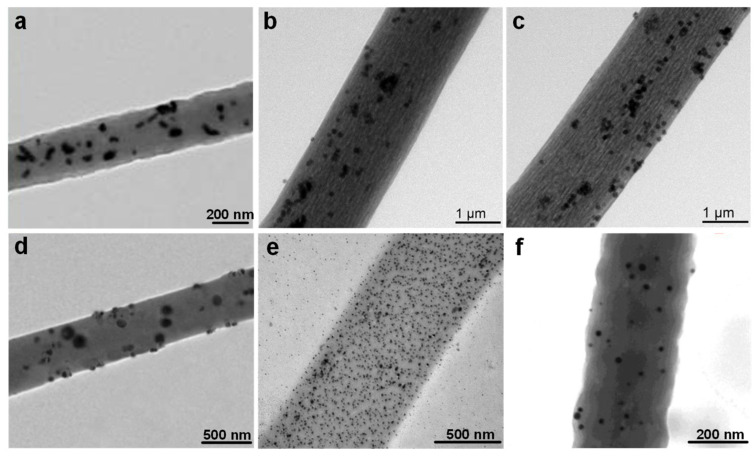
TEM images of electrospun composite polymer nanofibers. (**a**) PAN/Ag_2_CO_3_ [95]. (**b**) PS-Fe-Tb. (**c**) PVP-Fe-Tb [96]. (**d**) nTiO_2_/MET@MSNs-PCL/GEL NFs [97]. (**e**) Gelatin/Chitosan@AgNPs fiber [98]. (**f**) PAN-co-PAA@AuNPs [99].

To further enhance the triboelectric performance, durability, and functional integration of electrospun nanofibers, composite polymer nanofibers have attracted extensive attention. These are prepared by embedding functional nanomaterials such as metal nanoparticles, metal oxides, mesoporous structures, and rare earth complexes, into polymer matrices during the electrospinning process [93]. These hybrid systems offer enhanced surface charge densities, dielectric constants and interfacial polarization, and sometimes even additional properties such as biocompatibility, magnetism, or luminescence [94]. Representative TEM images of such composite nanofibers are shown in Figure 5a–f.

As depicted in Figure 5a, PAN/Ag_2_CO_3_ nanofiber mats display a relatively homogeneous dispersion of Ag_2_CO_3_ nanoparticles within the fiber interior, with only mild aggregation. Interestingly [100], the dispersion quality is closely related to the applied electrospinning voltage, as increased voltage from 14 kV to 22 kV enhances the electrostatic repulsion among nanoparticles, leading to better spatial distribution [101]. HR-TEM analysis further reveals that Ag_2_CO_3_ NPs are sheathed by polymer layers, which prevents agglomeration and corrosion while maintaining their crystalline structure. This internal encapsulation favors charge trapping and stability in triboelectric operation [95]. The presence of metal oxides like Ag_2_CO_3_ enhances the dielectric properties of the composite, improving charge generation and retention, while also contributing to higher stability and durability during long-term use in triboelectric applications. In Figure 5b,c, PS-Fe-Tb and PVP-Fe-Tb nanofibers incorporate rare earth-doped Fe nanoparticles within the polymeric matrix [102]. The presence of terbium-doped iron particles not only facilitates electron exchange at the interface but also potentially introduces magnetic or optical functionalities, making these materials suitable for multifunctional self-powered sensing platforms [96]. The addition of metal dopants such as Fe and Tb improves the electrical conductivity and triboelectric properties, leading to enhanced charge transfer efficiency and the ability to perform under more challenging environmental conditions.

Figure 5d presents nanofibers fabricated from polycaprolactone/gelatin (PCL/GEL) incorporating both mesoporous silica nanoparticles (MSNs) and nanoscale titanium dioxide (nTiO_2_). These inorganic fillers exhibit good interfacial compatibility with the biopolymer solution, leading to uniform dispersion without visible aggregation [103]. The well-dispersed MSNs and nTiO_2_ enhance dielectric polarization and mechanical integrity of the fiber network, which are essential for triboelectric charge separation and mechanical resilience [97]. Incorporating nTiO_2_ further boosts the material’s stability and long-term durability, preventing degradation under environmental stresses like moisture or temperature fluctuations. In Figure 5e, a gelatin/chitosan nanofiber system with embedded AgNPs is shown. TEM analysis confirms the successful integration of AgNPs into the fibrous matrix, with particle sizes ranging from 2 to 10 nm [104]. Although some aggregation is evident, the composite demonstrates enhanced conductivity and biocompatibility, suitable for wearable or implantable TENGs in healthcare applications [98]. The integration of AgNPs significantly improves the electrical conductivity of the nanofibers, enhancing charge transfer efficiency, while also contributing to the biocompatibility and stability of the TENG in biomedical environments. Finally, Figure 5f displays PAN-co-PAA nanofibers decorated with AuNPs. The AuNPs are evenly distributed on the fiber surface with diameters ranging from 6 to 26 nm [105]. This surface anchoring of noble metal nanoparticles significantly improves interfacial polarization and charge transfer efficiency, thereby contributing to high-performance triboelectric and sensing behaviors [99]. Noble metal nanoparticles like AuNPs further enhance the charge transfer efficiency by improving the conductivity at the interface and reducing energy losses during operation, thereby enhancing the overall performance and lifespan of the TENG.

These examples illustrate that composite nanofibers have obvious advantages over pure polymers, particularly in terms of the adjustability of dielectric properties, the improvement in mechanical strength, and the enhancement of charge storage and transfer capabilities. If researchers can carefully select functional nano-fillers, such as metal nanoparticles, metal oxides, or mesoporous structures, and uniformly disperse these fillers in the polymer matrix, the nanoscale structure and interface characteristics of the fibers can be precisely designed [64]. There exists a strong interfacial bond between the filler and the polymer. This strong interfacial bond ensures mechanical stability and promotes effective charge separation, which are crucial for the reliable and high-performance operation of the next generation of self-powered triboelectric sensing devices under actual environmental conditions [106].

To sum up, both pure electrospun and composite electrospun nanofibers exhibit excellent properties in triboelectric applications. Pure polymer fibers have the characteristics of simplicity and uniformity, while composite polymer fibers achieve multifunctionalization by adding nano-fillers. Appropriate material selection and engineering design can provide a universal platform for the electrical, mechanical and surface properties of custom TENG equipment [107]. To better compare the performance of different material systems, Table 2 summarizes key triboelectric parameters such as the surface charge density and dielectric constant under standardized test conditions [108].

## 4. Practical Application of Self-Powered Sensors Based on TENGs

Triboelectric nanogenerators (TENGs) have emerged as a groundbreaking energy-harvesting and sensing technology, enabling a new generation of self-powered systems across diverse application domains. By efficiently converting mechanical stimuli into electrical signals [113], TENGs eliminate the reliance on external power supplies and offer unprecedented opportunities for integrating sensing functionalities into flexible, wearable, and intelligent platforms. In recent years, extensive research efforts have been directed toward leveraging TENG-based sensors in real-world scenarios [4], particularly for intelligent human–machine interaction, health monitoring, voice recognition, and gesture control. These applications can be broadly classified into contact-type and non-contact-type systems, depending on the nature of the physical interaction involved. The following sections systematically review recent advances in both categories, highlighting the key functional mechanisms, innovative system architectures, and their practical significance in next-generation electronics and smart environments [114,115].

### 4.1. Contact-Type Applications

TENG-based self-powered sensors have achieved significant breakthroughs in the field of intelligent sensing, providing innovative and sustainable solutions for human–machine interaction and health monitoring. Their unique advantages including flexibility, high sensitivity, rapid response, and compatibility with various materials and device architectures, making them ideal for next-generation intelligent medical devices, robotics, and daily life systems. The following content focuses on the latest advances in contact-type applications, covering both human–machine interaction and health/motion monitoring [116,117].

#### 4.1.1. Contact-Based Human–Machine Interaction

Contact-based human–machine interaction (HMI) systems based on triboelectric nanogenerators (TENGs) have gained increasing attention due to their self-powered nature, fast responsiveness, and adaptability to various user interfaces. By harnessing electrospun nanofiber materials, these TENGs can be tailored to exhibit high sensitivity, flexibility, and functional integration, making them particularly suitable for interactive systems where touch or mechanical contact is the primary mode of input [118].

**Figure 6:**
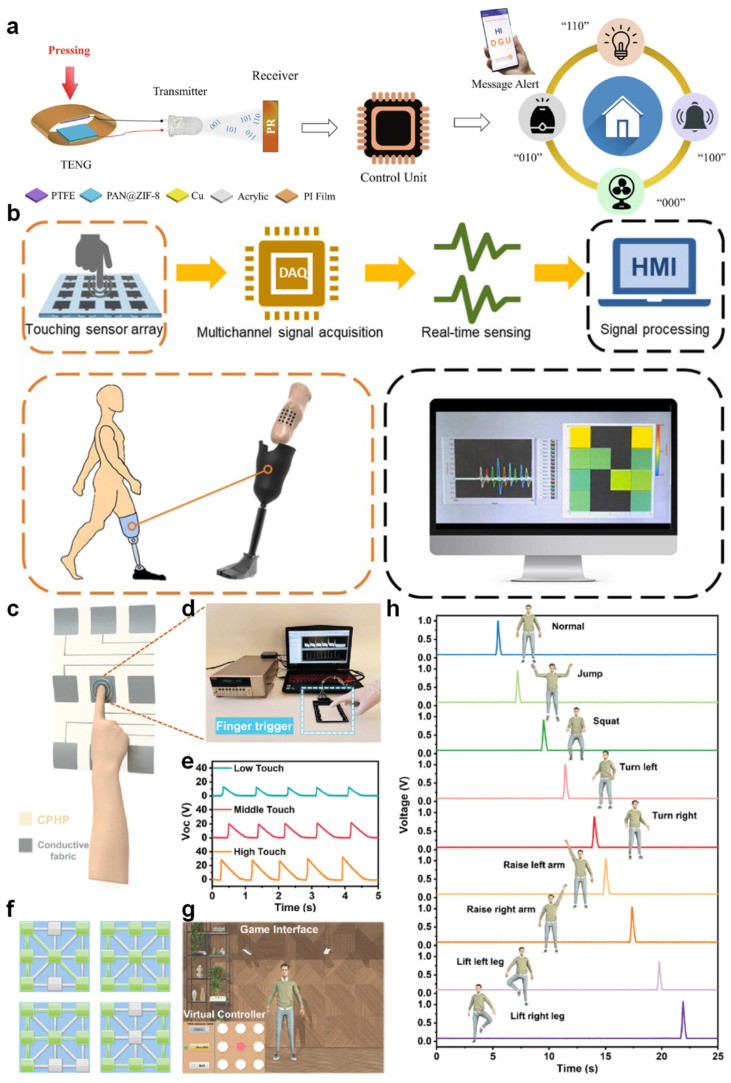
Contact HMIs based on electrospun TENGs. (**a**) Schematic of a self-powered VLC system using PZ-TENG for wireless signal transmission [121]. (**b**) Real-time prosthetic pressure monitoring using a TENG sensor array and DAQ-HMI interface [128]. (**c**) Structure of a 3 × 3 CPHP-based sensing matrix. (**d**) Experimental setup demonstrating finger-triggered voltage output. (**e**) Voltage signals under varied pressure intensities for binary tactile encoding. (**f**) Response of the matrix indicator lights based on LabVIEW, triggered by CPHP-TS. (**g**) Game character interface based on LabVIEW, using CPHP-TS as the controller. (**h**) Output voltage signals of CPHP-TS and the corresponding character movement commands [130].

Pandey et al. proposed a wireless communication platform that employs a piezoelectric–triboelectric hybrid nanogenerator (PZ-TENG) to drive a visible light communication (VLC) system [119]. Their work demonstrated that mechanical stimuli, such as pressing or flapping, can be directly converted into modulated optical signals via LEDs without an external power supply [120]. The signals are subsequently received by a photodetector and decoded by a microcontroller unit (MCU), enabling real-time transmission of 3-bit logic codes [121,122]. This architecture allows for diverse applications such as wireless control of home appliances and secure message transmission [123]. Their design showcases the potential of TENGs in compact, energy-autonomous HMI systems where physical actions are translated into digital commands via optical signaling (Figure 6a) [124]. In a separate study, Chang et al. developed a tactile sensor array based on TENGs for real-time pressure mapping within prosthetic sockets [125]. The array was integrated with a multichannel data acquisition (DAQ) system to spatially resolve inner-socket pressure during use [126]. Through a human–machine interface (HMI) display, users could visually interpret pressure distributions in real time, which facilitates adjustments for personalized prosthesis fitting [127]. Their system, as illustrated in Figure 6b, emphasizes the applicability of TENG-enabled pressure sensors in biomedical and rehabilitation settings, where dynamic mechanical feedback is essential for user comfort and device adaptation [128,129].

Further expanding the functionality of triboelectric HMI, Niu et al. reported a capacitive–triboelectric hybrid paper (CPHP) sensor composed of a 3 × 3 array of electrospun sensing units. Each unit, consisting of a triboelectric layer and a conductive electrode, responded sensitively to finger tapping with varying intensities. The electrical outputs, proportional to the applied pressure, were interpreted via a signal processing circuit and digitized for further application. Notably, the system could distinguish low-, medium-, and high-force inputs and use them for encoding information akin to Morse code. For instance, different touch intensities were employed to encode the letters in “TENG”, “SENSOR” and other terms, thereby providing a low-energy, tactile communication mechanism (Figure 6c–e) [130,131]. Beyond static touch recognition, the CPHP interface demonstrated capabilities for trajectory-based interaction. By tracking the activation sequence of sensing units during sliding motions, Niu’s system was able to reconstruct finger paths and display them as spatially accurate patterns on a LabVIEW-controlled interface. Such dynamic feedback is critical for applications in smart surfaces and virtual writing systems, where real-time recognition of gesture trajectories is required (Figure 6f). The sensor exhibited minimal signal crosstalk between adjacent channels, reflecting its high resolution and signal integrity [132].

In addition, the same sensing matrix was employed to control a virtual avatar in a simulated gaming environment. Each sensing unit in the CPHP array was mapped to a distinct command such as jumping, squatting, turning, or limb lifting, and the corresponding output voltages were used to trigger actions once exceeding a pre-set threshold (Figure 6g,h). This real-time control system demonstrates that it is feasible to use TENG-based tactile sensors as energy-autonomous game controllers or as auxiliary devices based on gesture control. These studies together show that TENGs based on electrospun nanofibers have made progress in promoting the development of contact-mode human–machine interfaces. With innovations in architecture, integration of signal processing, and multifunctional sensing design, these systems can provide energy sustainability and offer rich interactive experiences, ranging from biomedical prosthetic feedback to gestured virtual environments. The role of TENGs is no longer merely that of a pure energy collector. Instead, it has transformed into a multifunctional interactive node capable of intelligent perception and communication. The continued optimization of nanofiber morphology, triboelectric material pairing, and electronic interface design is expected to further broaden the applicability of these systems across wearable electronics, soft robotics, and next-generation user interfaces [133].

#### 4.1.2. Contact-Based Health and Motion Monitoring

With the appearance of wearable electronic products, TENGs based on electrospun nanofibers will be the best choice for real-time health and motion monitoring systems that can track people’s mechanical activities continuously without being powered by an external source. Therefore, they are very attractive for long-term physiological detection and gait identification [134].

**Figure 7:**
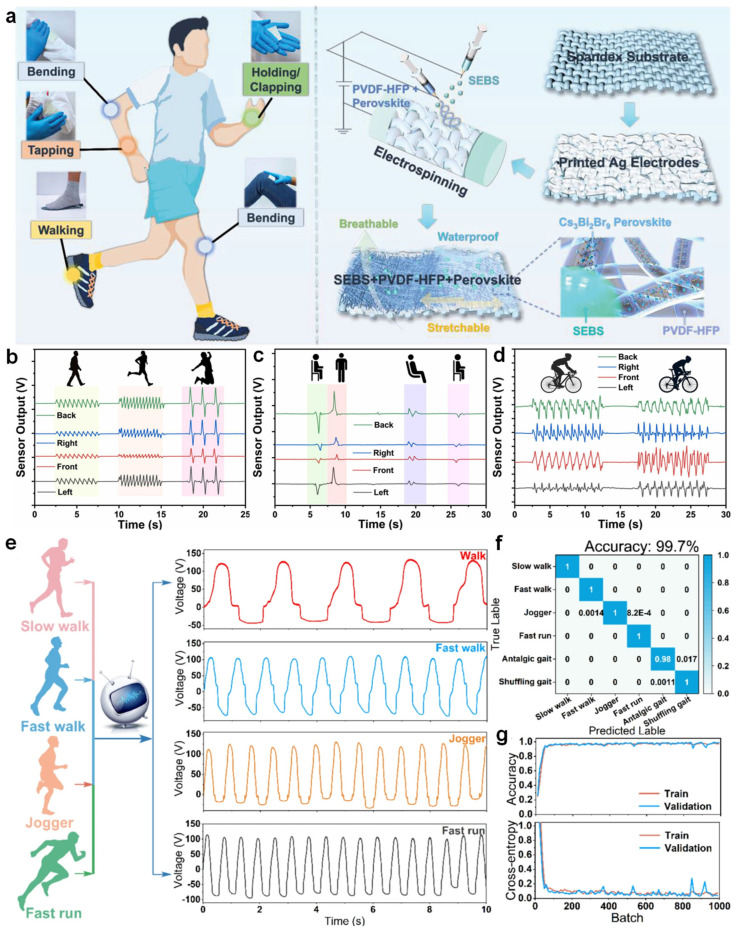
Contact-based health and motion monitoring using electrospun TENGs. (**a**) Hybrid nanofiber TENG integrated on textile for motion energy harvesting [136]. (**b**) Depiction of energy harvesting during walking, running, and jumping movements. (**c**) Monitoring transitions such as sitting down to standing up, and shifting from upright sitting to a reclined position. (**d**) Cycling scenarios illustrating posture detection for both standard and non-standard seating positions [138]. (**e**) Output voltage profiles corresponding to various movement types, including slow walking, brisk walking, jogging, and sprinting. (**f**) Deep learning-derived confusion matrix illustrating the classification accuracy for different gait patterns. (**g**) Performance curve showing the training progress of the 1D-CNN model used for signal classification [139].

Jiang et al. developed a stretchable and waterproof TENG by employing a dual-needle electrospinning technique to fabricate composite nanofibers composed of PVDF-HFP, SEBS, and Cs_3_Bi_2_Br_9_ perovskite [135]. The nanofiber layer was integrated with breathable spandex fabric and screen-printed Ag electrodes to form a conformable sensor [126]. When attached to various body joints, the device could harvest motion-induced energy during activities such as walking, tapping, or joint bending, showing excellent mechanical durability and signal stability under continuous deformation (Figure 7a) [136]. To assess motion states, Faruk and co-workers developed a smart belt incorporating TENG units, capable of detecting a wide range of physical actions including walking, running, jumping, sitting, and posture transitions [137]. The generated voltage signals exhibited distinguishable waveform features in both amplitude and frequency domains, enabling accurate differentiation between dynamic gaits and subtle postural shifts. For instance, characteristic signal reversals during sitting or standing transitions allowed reliable temporal mapping of behavioral changes, while irregular signal profiles during cycling reflected abnormal posture conditions (Figure 7b–d) [135,138].

Building on high-resolution signal acquisition, Hao et al. designed a triboelectric sensing textile for gait recognition and applied a one-dimensional convolutional neural network (1D-CNN) to classify six locomotion patterns, including pathological gaits such as shuffling and antalgic walking [140]. They collected triboelectric voltage signals from the wearer’s heel and achieved a recognition accuracy of 99.7%, with minimal overfitting across individuals, and strong robustness. Their approach effectively captured subtle spatiotemporal features embedded within complex motion patterns by leveraging an artificial intelligence algorithm (Figure 7e–g) [139,141]. These accomplishments collectively demonstrate the usefulness of electrospun TENGs in worn/contact-type health monitoring applications as a synergistic platform for self-powered sensing, biomechanics study, and real-time behavioral recognition.

In summary, TENG-based self-powered sensors have demonstrated outstanding advantages and broad application prospects in contact-type intelligent sensing [24]. Their integration into human–machine interaction and health/motion monitoring systems enables more efficient, real-time, and intelligent solutions [120]. As these technologies continue to evolve and are combined with wearable electronics and artificial intelligence, they are expected to play an increasingly important role in personalized healthcare, smart environments, and next-generation interactive technologies [142].

### 4.2. Non-Contact-Type Applications

In recent years, contactless technologies have attracted considerable attention across multiple fields due to their advantages in hygiene, accessibility, and user-friendliness [143]. Within the domain of triboelectric nanogenerators (TENGs), contactless applications have rapidly expanded, particularly in the areas of voice recognition and human–machine interaction (HMI). By leveraging their ability to harvest and convert mechanical or acoustic stimuli into electrical signals [144], TENG-based systems offer promising solutions for developing self-powered, low-latency, and wearable platforms. Notably, contactless voice recognition systems integrated with TENGs enable accurate speech-to-text conversion without the need for traditional microphones, facilitating applications in smart assistants, security authentication, and assistive communication. Simultaneously, TENG-driven HMI interfaces allow users to control devices or communicate with machines through gestures [145], proximity, or speech, establishing a new paradigm for seamless, non-invasive interaction between humans and intelligent systems. This section reviews representative studies that demonstrate the innovative use of TENGs in contactless voice recognition and HMI, highlighting both the functional mechanisms and their practical implications [146].

#### 4.2.1. Non-Contact Human–Machine Interaction

**Figure 8:**
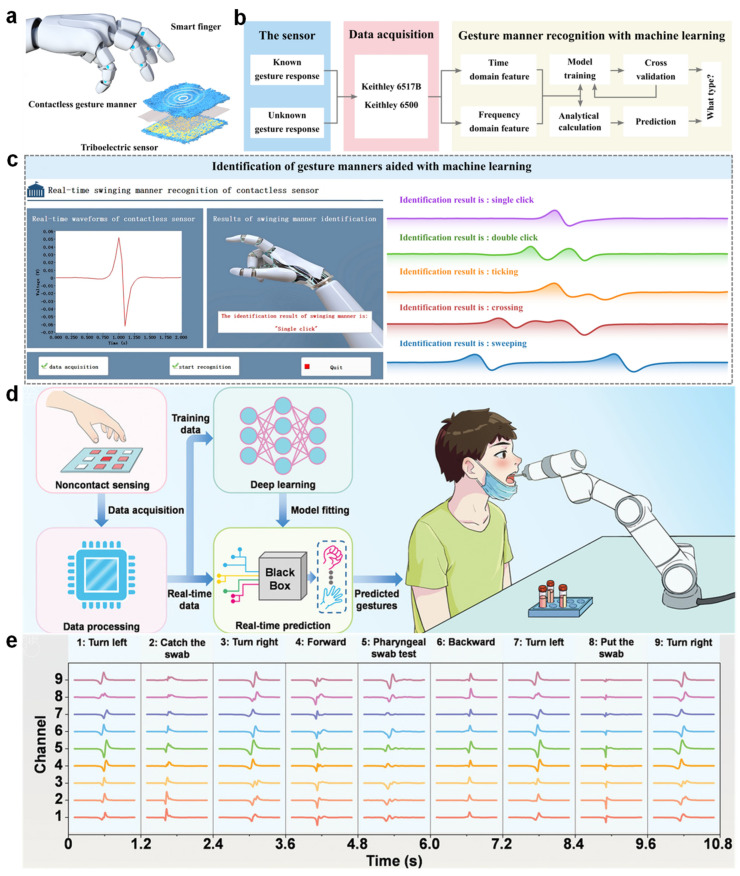
Non-contact HMIs based on electrospun TENGs. (**a**) Illustration of a TENG-based sensor-enabled system for recognizing contactless gesture manners. (**b**) Schematic diagram of the data acquisition, processing, and machine learning-based gesture classification pipeline [150]. (**c**) Real-time demonstration interface of contactless gesture recognition, showing voltage signal acquisition and gesture prediction results. (**d**) Workflow of a deep learning-assisted, non-contact gesture recognition system applied to robotic throat swab sampling, highlighting signal acquisition, model prediction, and robot control. (**e**) Representative multichannel gesture signals used to guide robotic actions during the sampling process, enabling contactless medical operations [151].

To extend the usability of triboelectric nanogenerators (TENGs) beyond physical interfaces, recent studies have demonstrated their potential in recognizing contactless gestures [147]. For instance, Ye et al. developed a contactless TENG-based sensor system capable of identifying multiple gesture types without direct touch, thus enabling a new form of human–machine interaction (Figure 8a). In their work, various gesture-induced electrostatic signals were generated by moving a PTFE stick in proximity to the sensor, covering actions such as single click, double click, ticking, crossing, and sweeping. Although the resulting signal waveforms appeared highly similar, the incorporation of machine learning algorithms effectively differentiated between them [148,149].

The gesture recognition process was structured into three main phases: signal acquisition [144], feature extraction, and classification (Figure 8b). Time-domain and frequency-domain features were extracted from repeated measurements of each gesture (collected 100 times), which were then used to train various classification models. Principal component analysis (PCA) revealed distinguishable clustering in a two-dimensional feature space, laying the foundation for accurate gesture categorization [152]. Among the tested algorithms including KNN, decision trees, support vector machines, multilayer perceptrons, random forests, and XGBoost, the highest classification accuracy reached 98%, highlighting the suitability of ensemble-based approaches for this task [150,153]. To further demonstrate its practical applicability, a real-time gesture recognition platform was developed based on the trained random forest model (Figure 8c). This system enabled live signal visualization and immediate feedback on recognized gestures, allowing users to interact with the machine in a non-contact and intuitive manner [147]. Such integration of triboelectric sensing and intelligent data processing offers promising opportunities for next-generation human–machine interfaces in scenarios requiring hygienic, noninvasive, or remote operation [154].

Beyond conventional gesture-based interfaces, triboelectric nanogenerator-based systems have also shown promise in intelligent medical applications requiring hygienic, non-contact interaction. Zhou et al. proposed an advanced non-contact gesture recognition framework that integrates triboelectric tactile sensors (TTS) with deep learning to facilitate remote control of robotic systems for medical tasks, such as automated throat swab sampling during infectious disease outbreaks (Figure 8d) [119]. The proposed system employs a multichannel TTS array capable of capturing spatially distributed electrostatic signals generated by hand gestures performed above the sensing surface [155]. These signals are processed in real time and analyzed using a multilayer perceptron (MLP) neural network trained to associate gesture patterns with predefined commands. As illustrated in Figure 8e, nine distinct gestures such as turning left, grabbing the swab, or executing a forward motion can be accurately recognized and translated into corresponding robotic actions [156]. Notably, this intelligent interface enables medical personnel to guide the robot through complex sampling sequences entirely without physical contact, thus minimizing the risk of cross-infection in high-risk environments [151,157].

Moreover, the system demonstrated strong robustness under varying electromagnetic noise levels and mechanical strain conditions, maintaining high recognition accuracy, even when the TTS array was stretched up to 150%. This adaptability highlights the potential of TENG-based non-contact interfaces in practical clinical settings where flexibility and reliability are essential [158].

#### 4.2.2. Non-Contact Voice Recognition

**Figure 9:**
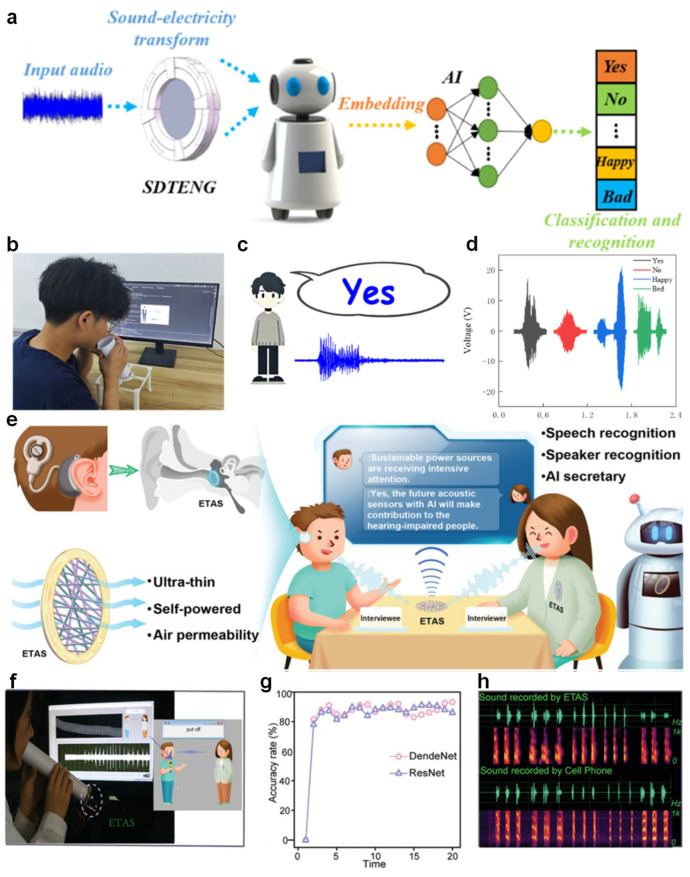
Non-contact voice recognition. (**a**) Schematic illustration of the SDTENG-enabled human–machine interface for sound-to-electricity conversion and speech recognition. (**b**) Experimental demonstration of speech input using the SDTENG-based system. (**c**) Output interface showing recognition results of spoken words. (**d**) Voltage waveforms corresponding to different voice commands recorded by the SDTENG [162]. (**e**) Concept of ETAS integration with hearing aids to facilitate communication for hearing-impaired individuals. (**f**) Real-time voice-to-text conversion enabled by the ETAS module. (**g**) Model performance comparison between DenseNet and ResNet in terms of voice recognition accuracy. (**h**) Comparison of audio waveforms and spectrograms recorded by the ETAS and a conventional cell phone [163].

In the context of non-contact human–machine interaction, voice recognition technologies are gaining increasing attention due to their intuitive usability and contactless nature [159,160]. Dai et al. proposed a bioinspired speech recognition system integrating a sound-driven triboelectric nanogenerator (SDTENG) capable of converting acoustic signals into electrical outputs for further processing (Figure 9a). This system leverages embedded feature extraction and AI-based classification algorithms to accurately recognize different voice commands and emotional expressions [161].

During experimental validation, as shown in Figure 9b, the user vocalizes a word near the SDTENG device, and the processed signal is promptly displayed on a recognition interface [164]. The design of the interface allows for immediate visualization of the classification result (Figure 9c). Moreover, the electrical signal waveforms generated in response to different spoken words such as “Yes”, “No”, “Happy”, and “Bad” exhibit distinct patterns (Figure 9d), demonstrating the system’s effectiveness in distinguishing between various vocal inputs. This work highlights the potential of TENG-based systems in developing energy-efficient, real-time, and contact-free voice-controlled applications [162,165]. In recent advances aimed at enhancing voice recognition accessibility, Jiang et al. developed an innovative epidermal triboelectric acoustic sensor (ETAS) designed for seamless integration with the human body [166], offering a flexible and self-powered interface for acoustic signal acquisition (Figure 9e). This technology addresses key challenges faced by individuals with hearing impairments, enabling them to participate in real-time verbal interactions and even perform tasks such as interviews through voice-to-text conversion [167]. The system captures acoustic signals through the ETAS module, which are then processed using a convolutional neural network based on DenseNet architecture [168].

In practical demonstrations, the user speaks into the ETAS-enabled system, where the audio signals are promptly captured and processed for recognition (Figure 9f). The recorded acoustic features are processed through a deep learning pipeline based on DenseNet and compared with ResNet for performance evaluation. As shown in Figure 9g, DenseNet achieves superior classification accuracy over 20 training epochs, outperforming ResNet with a final test accuracy of 93.25% versus 86%, respectively. Furthermore, Figure 9h illustrates a comparative waveform and spectrogram analysis between ETAS and conventional mobile phone recordings [169]. The ETAS-captured signals exhibit cleaner frequency components and temporal resolution, confirming the system’s efficacy in precise voice capture. This combination of flexible sensing and robust neural inference demonstrates a promising direction for non-invasive and real-time speech recognition technologies [163].

Overall, contactless applications enabled by TENG technology present a transformative approach to achieving intuitive and responsive interaction between users and machines [170]. The integration of TENG-based acoustic sensors, such as SDTENG and ETAS, into AI-enhanced frameworks has demonstrated significant potential in recognizing voice commands and emotional tones with high precision, even in complex environments [171]. Meanwhile, contactless HMI systems offer expanded functionalities beyond speech, such as gesture-triggered operations or proximity-sensitive controls [172], broadening the applicability of TENGs in fields ranging from healthcare to consumer electronics. Future research is expected to focus on improving system robustness, miniaturization, and multi-modal sensing to further enhance real-world deployment. As a result, TENG-driven contactless technologies are poised to play a critical role in the evolution of smart, accessible, and interactive electronics [173,174].

## 5. Conclusions and Future Perspectives

In summary, electrospun nanofiber-based self-powered TENGs have exhibited remarkable potential in the development of next-generation intelligent sensing technologies, particularly in both contact and non-contact sensing domains. Through the integration of innovative device architectures, advanced material engineering, and electrospinning fabrication strategies, these sensors possess numerous advantageous characteristics, including high sensitivity, mechanical flexibility, lightweight structure, excellent biocompatibility, and the ability to generate energy autonomously. These features not only enable TENG-based sensors to operate independently without external power sources but also make them highly suitable for wearable, portable, and miniaturized applications. Their versatile utility has been demonstrated in diverse fields such as human–machine interfaces, real-time health and motion monitoring, gesture and tactile sensing, and intelligent voice recognition systems, offering sustainable, low-power, and multifunctional sensing platforms.

The use of electrospun nanofibers as triboelectric active layers provides a unique and tunable pathway to manipulate sensor performance at the nanoscale, enabling precise control over surface morphology, dielectric properties, and interfacial charge dynamics. By employing pure or composite polymer nanofibers combined with flexible or stretchable substrates, the mechanical adaptability, electrical output, and environmental durability of TENGs can be significantly enhanced. This synergy facilitates the development of intelligent electronic skins, soft robotics, implantable biomedical sensors, and multifunctional diagnostic tools. With continued progress in materials science, nanomanufacturing, and system integration, electrospun nanofiber-based TENGs are expected to play an increasingly vital role in the advancement of smart, autonomous, and human interactive electronic systems.

Looking ahead, several critical challenges must be overcome to advance the practical applications of electrospun nanofiber-based TENG sensors. Firstly, it is crucial to address long-term operational stability and durability under dynamic environmental conditions, such as temperature fluctuations, humidity, and mechanical fatigue. Issues such as material degradation, charge saturation, and fatigue in polymer-based TENGs significantly hinder their commercialization. Additionally, the sensitivity to environmental factors like humidity and mechanical wear must be mitigated to ensure consistent performance over extended periods of use. Secondly, it is imperative to develop scalable, low-cost, and reproducible fabrication methods to facilitate large-scale manufacturing and commercial adoption. Thirdly, the integration of TENG-based sensors with artificial intelligence (AI), wireless communication, and Internet of Things (IoT) technologies will be essential for building intelligent systems capable of autonomous decision-making, real-time data processing, and remote monitoring.

To address these challenges, future research should focus on exploring novel materials and mechanisms that can enhance the performance and longevity of TENGs. For example, incorporating self-healing, recyclable, or biodegradable materials could improve the sustainability and operational stability of these sensors in real-world environments. Furthermore, developing hybrid sensing platforms that combine triboelectricity with other energy harvesting mechanisms, such as piezoelectricity or thermoelectricity, could offer solutions to low energy conversion efficiency and broaden the functionality of TENG-based systems. These innovations can lead to next-generation devices with enhanced performance, broader application scopes, and improved user safety. With continuous progress in nanomaterial synthesis, structural design, and system-level integration, electrospun nanofiber-based TENG sensors are expected to play a transformative role in the future of self-powered intelligent sensing. Their potential in healthcare, environmental surveillance, wearable electronics, and soft robotics offers a promising pathway toward smart, energy-efficient, and sustainable electronic systems.

## Figures and Tables

**Table 1:** Comparison of electrospinning-based, MXene-based, and polymer blend-based materials for TENGs.

Material	Advantages	Challenges
Electrospinning-based	High surface area, tunable fiber morphology	Scaling up, fiber uniformity, mechanical stability
MXene-based	High conductivity, mechanical strength, flexibility	Synthesis complexity, material cost
Polymer blend-based	Improved mechanical properties, charge generation	Blending compatibility, processing difficulty

**Table 2:** Comparative summary of triboelectric parameters for different material systems.

Material System	Surface Charge Density (μC/m^2^)	Dielectric Constant	Reference
PVDF	8.5	10	[109]
PAN	6.3	7.5	[110]
PCL	7.1	6.0	[111]
PAN/Ag_2_CO_3_	10.2	12.5	[112]
PCL/GEL (MSNs & nTiO_2_)	9.8	13.0	[97]

## Data Availability

Data sharing is not applicable.
